# Fluid geochemistry, local hydrology, and metabolic activity define methanogen community size and composition in deep-sea hydrothermal vents

**DOI:** 10.1038/s41396-019-0382-3

**Published:** 2019-03-06

**Authors:** Lucy C. Stewart, Christopher K. Algar, Caroline S. Fortunato, Benjamin I. Larson, Joseph J. Vallino, Julie A. Huber, David A. Butterfield, James F. Holden

**Affiliations:** 1 Department of Microbiology, University of Massachusetts, Amherst, MA 01003 USA; 2 Department of Oceanography, Dalhousie University, Halifax, B3H 4R2 Canada; 3 Department of Biology, Wilkes University, Wilkes-Barre, PA 18766 USA; 4 Joint Institute for the Study of Atmosphere and Ocean, University of Washington, Seattle, WA 98195 USA; 5 Ecosystems Center, Marine Biological Laboratory, Woods Hole, MA 02543 USA; 6 Marine Chemistry and Geochemistry, Woods Hole Oceanographic Institution, Woods Hole, MA 02543 USA; 7 Present Address: GNS Science, Wellington, 5010 New Zealand

**Keywords:** Biogeochemistry, Biogeochemistry, Archaea, Water microbiology

## Abstract

The size and biogeochemical impact of the subseafloor biosphere in oceanic crust remain largely unknown due to sampling limitations. We used reactive transport modeling to estimate the size of the subseafloor methanogen population, volume of crust occupied, fluid residence time, and nature of the subsurface mixing zone for two low-temperature hydrothermal vents at Axial Seamount. Monod CH_4_ production kinetics based on chemostat H_2_ availability and batch-culture Arrhenius growth kinetics for the hyperthermophile *Methanocaldococcus jannaschii* and thermophile *Methanothermococcus thermolithotrophicus* were used to develop and parameterize a reactive transport model, which was constrained by field measurements of H_2_, CH_4_, and metagenome methanogen concentration estimates in 20–40 °C hydrothermal fluids. Model results showed that hyperthermophilic methanogens dominate in systems where a narrow flow path geometry is maintained, while thermophilic methanogens dominate in systems where the flow geometry expands. At Axial Seamount, the residence time of fluid below the surface was 29–33 h. Only 10^11^ methanogenic cells occupying 1.8–18 m^3^ of ocean crust per m^2^ of vent seafloor area were needed to produce the observed CH_4_ anomalies. We show that variations in local geology at diffuse vents can create fluid flow paths that are stable over space and time, harboring persistent and distinct microbial communities.

## Introduction

**Fig. 1:**
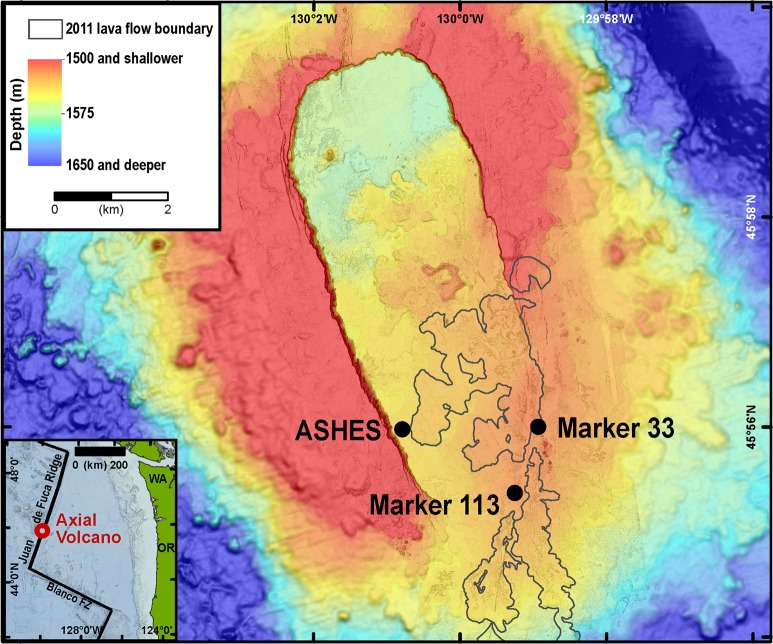
Map of Axial Seamount and the sampling locations. The hydrothermal sampling sites were along the southeastern rim of the caldera. The outline of the 2011 lava flow is from Caress et al. [42]. The inset shows the location of Axial seamount in the NE Pacific Ocean. The map is based on MBARI autonomous underwater vehicle bathymetry (2 m grid) overlain on shipboard multibeam bathymetry (20 m grid)

The igneous ocean crust contains 2% of the fluid volume of the overlying global ocean [1] and an estimated 1.5 Pg of microbial carbon [2]. Diverse chemolithotrophic *Epsilonbacteraeota* living in the global ocean crust at deep-sea hydrothermal vents contain an estimated 1.4–2.7 Gg C and produce 0.045–1.4 Tg of organic C yr^−1^, suggesting that microbes in the shallow hydrothermal subseafloor are a relatively small standing stock which turns over rapidly [3]. However, quantifying the biogeochemical impact of subseafloor microbes in the anoxic zones of oceanic crust remains a challenge. At Axial Seamount in the northeastern Pacific Ocean (Fig. 1), low-temperature (<50 °C) diffuse hydrothermal fluids steadily emanate from cracks in the basaltic seafloor and contain biogenic CH_4_ and culturable methanogenic microbes [4–8]. Both molecular and culture-based analyses of these fluids showed that the predominant methanogens present are thermophilic *Methanothermococcus* and hyperthermophilic *Methanocaldococcus* species [6, 9–12]. They are among the most common high-temperature methanogens found globally in hydrothermal vents [13–15] and hot subsurface petroleum reservoirs [16–19].

Communities of methanogens and other microbes at individual vents at Axial Seamount are distinct and persistent over time, demonstrating that the subseafloor habitat consists of stable localized microbial communities that reflect their local environment [12, 20, 21]. However, the inaccessibility of the igneous subsurface makes it difficult to measure the total number of active methanogens that inhabit the subseafloor at each vent site, the environmental factors shaping the composition of these microbial communities, and their biogeochemical impact on the diffuse fluids exiting the seafloor into the overlying ocean. One approach for quantitatively assessing these questions is reactive transport modeling. However, developing models of subsurface microbial growth relies on correctly parameterizing the growth kinetics of the resident microbes. The *Methanothermococcus* and *Methanocaldococcus* species dominant at Axial grow primarily by combining 4H_2_ and CO_2_ to produce CH_4_ and 2H_2_O. Microcosm incubations of diffuse vent fluids from Axial Seamount demonstrated that methanogenesis at 55 °C and 80 °C does not use formate or acetate and was limited by H_2_ availability rather than anabolic needs such as nitrogen, vitamins, or trace metals [8]. Therefore, these two genera of methanogens are ideal for reactive transport modeling using H_2_ concentration and temperature as the primary variables.

To predict the minimum threshold of H_2_ needed for growth, three *Methanocaldococcus* spp. were grown previously in a gas-purged batch reactor at different H_2_ concentrations to determine their H_2_ Monod kinetics for growth [6]. This threshold predicted the presence or absence of hyperthermophilic methanogens in various global hydrothermal systems based on H_2_ availability. However, a chemostat is necessary to estimate CH_4_ production rates by methanogens at different H_2_ concentrations. In this study, CH_4_ production rates and Monod kinetics at varying H_2_ concentrations were determined using a chemostat for *Methanocaldococcus jannaschii* and *Methanothermococcus thermolithotrophicus*. Arrhenius growth constants at varying temperatures were also determined using Balch tubes. These data were used to parameterize a reactive transport model describing the growth and transport of *M. jannaschii* and *M. thermolithotrophicus* beneath the seafloor, along with H_2_, heat, and Mg^2+^, from the high-temperature source fluid and diluted with seawater until the fluid reached the temperature observed on the seafloor. The model was applied to two physically distinct deep-sea hydrothermal vent sites at Axial Seamount. This work highlights the potential of coupling pure culture laboratory studies of environmental processes with reactive transport modeling and field observations to determine the size and nature of biogeochemical processes in environments that are otherwise difficult to access.

## Methods

### Chemostats

*Methanocaldococcus jannaschii* DSM 2661 [22] and *Methanothermococcus thermolithotrophicus* DSM 2095 [23] were purchased from the Deutsche Sammlung von Mikroorganismen und Zellkulturen GmbH (DSMZ, Braunschweig, Germany). For chemostat growth, *M. jannaschii* was grown at 82 and 65 °C and *M. thermolithotrophicus* was grown at 65 and 55 °C in a 2-L bioreactor with a working volume of 1.5 L. The growth medium was modified DSM 282 medium [22]. The medium was reduced with 0.025% each of Na_2_S•9H_2_O and cysteine-HCl, stirred at 300 rpm, and sparged with 7.5 mL min^−1^ CO_2_ and varying flow rates of H_2_ and N_2_ to bring the total gas flow rate to 70 mL min^−1^, or to 100 mL min^−1^ for the highest H_2_ concentrations. The medium was maintained at pH 6.0 ± 0.1 by the automatic addition of 0.25 mM HCl.

The organisms were grown in batch phase prior to initiating the chemostat until they reached late logarithmic growth phase. The growth medium in the bioreactor was replaced with fresh sterile medium from an 18.5 L reservoir that was sparged with N_2_ and heated to the same temperature as the bioreactor. The dilution rate of the chemostat matched the batch-phase growth rate of the organism for the temperature and H_2_ concentration provided until steady-state conditions were reached (assumed to be after three full replacements of the medium based on longer test runs). The chemostat was operated such that all the H_2_ input into the reactor was consumed by the cells and the H_2_ output concentration was below the level of detection. Cell concentrations were determined using a Petroff-Hausser counting chamber and phase-contrast light microscopy. The headspace gas of the bioreactor was sampled directly using a Hamilton gas-tight syringe through a septum. The gas concentrations in the liquid medium were measured by anoxically transferring 20 mL of medium into a sealed 60 mL serum bottle that was pre-flushed with N_2_, allowing the gases to equilibrate into the headspace. The concentration of CH_4_ in the headspace was measured using a gas chromatograph (Shimadzu GC-17A) with a flame-ionization detector and a molecular sieve 5A column at 120 °C. The concentration of H_2_ was also measured using a gas chromatograph (Shimadzu GC-8A) with a thermal conductivity detector and an Alltech Haysep DB 100/120 column at 120 °C. The gas flow rate into the bioreactor was measured using a bubble meter. The cell-specific CH_4_ production rate was calculated from the sum of the CH_4_ concentration in the headspace times the gas flow rate and the CH_4_ concentration in the medium times the medium dilution rate, which was normalized by the total cell concentration in the reactor. Each measurement was taken in duplicate, and the dilution rate was changed midway through each chemostat run to determine the effect of changing the growth rate of the organisms on the rate of CH_4_ production.

To determine the effect of temperature on growth, growth rate was determined for cells incubated between 84 and 45 °C for *M. jannaschii* and between 68 and 30 °C for *M. thermolithotrophicus*. Cells were grown in modified DSM 282 medium as described above in Balch tubes sealed with butyl rubber stoppers and containing 2 atm (100 kPa added pressure at room temperature) of H_2_:CO_2_ (80%:20%) headspace. At various time points during growth, the cell concentration in the tubes was determined as described above. The growth rate was determined from a best-fit curve through the exponential portion of cell growth. The 95% confidence interval of the growth rate was determined using analysis of covariance (ANCOVA) [24].

### Model description

A major challenge in developing the reactive transport model for methanogen growth is the lack of information on the size and configuration of the subsurface hydrothermal mixing zone. While numerical models using Darcy flow through a porous medium have captured the dominant features of hydrothermal circulation over the scale of an entire vent field or ridge system (i.e., 1–10 km) [25, 26], such models lack the resolution necessary to describe fluid flow at the scale of an individual vent, where flow is most likely controlled by the unique configuration of cracks and fissures feeding an outflow [27, 28]. Therefore, we took a simpler approach, whereby the flow was parameterized (rather than modeled using Darcy flow), but mass conservation was maintained.

**Fig. 2:**
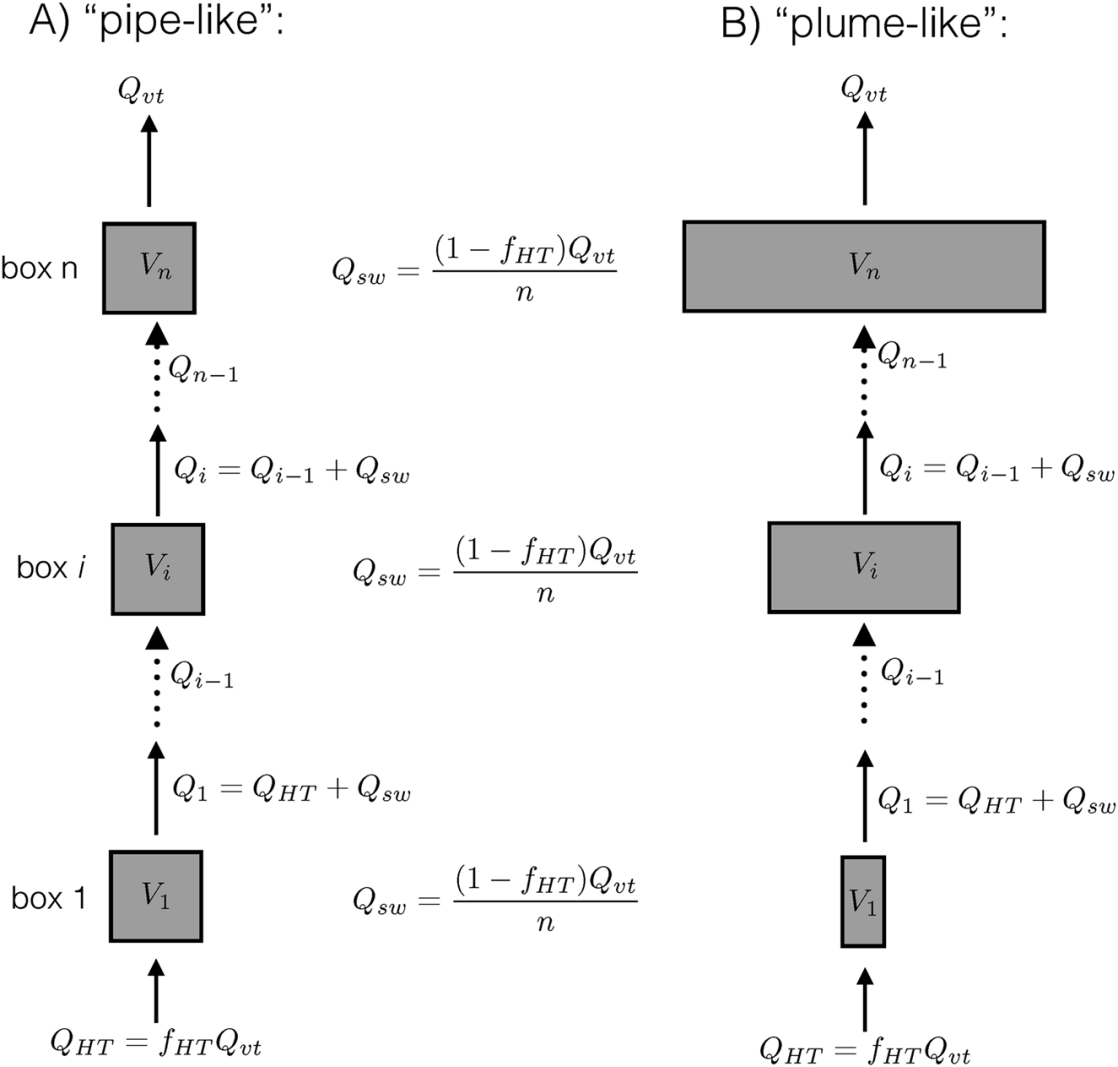
Model geometry and transport for **a** a straight-pipe reactive transport model (*x*_b_ >> 1) and **b** an expanding-plume reactive transport model (*x*_b_ << 1)

The model describes the growth of methanogens in a high-temperature end-member fluid devoid of Mg^2+^ that is transported along a flow path and progressively diluted with seawater until it exits the seafloor. To represent fluid transport, the model considered a one-dimensional flow path consisting of a series of *n* boxes (Fig. 2). High-temperature hydrothermal fluid, lacking Mg^2+^, entered the first box with the fluid composition of the high-temperature end-member, then flowed from box-to-box, and at each box was progressively diluted with an equal amount of 2 °C seawater. Because the true length of the flow path is unknown, the model is non-dimensionalized with respect to space, such that the sum of all the box volumes is equal to one and represents the total volume of the subsurface mixing zone feeding the vent outflow. The total residence time of fluid in the system is set by the spatially non-dimensionalized fluid flux exiting the seafloor (*Q′*_*vt*_), which has units of time^−1^ and can be thought of as a dilution factor that defines the timescale of hydrothermal fluid circulation. The residence time fluid spends at different temperatures along the flow path is controlled by both *Q′*_*vt*_ and the volume of each individual box along the flow path. To simplify the specification of box volumes, the volume of each box is given by the following formula:

1 $$\Delta V_i = \frac{1}{n} \cdot \frac{{e^{x_b} \cdot e^{\frac{{x - 1}}{{x_b}}}}}{{x_b \cdot (e^{x_b} - 1)}}$$

where *V* is the box volume, *n* is the total number of boxes, *x* is a non-dimensional variable describing the position along the flow path and varies from 0 at the high-temperature end-member to 1 at the point where fluid is venting into the deep ocean, and *x*_b_ is a shape parameter describing the geometry of the subsurface mixing zone. The shape parameter allowed the model to transition between two different mixing regimes. If *x*_b_ >> 1, then the flow path resembled a linear crack or straight pipe (Fig. 2a). If *x*_b_ << 1, then the flow path spread out as it rose, approximating an expanding plume percolating through the ocean crust (Fig. 2b). The fluid flux, *Q′*_*vt*_, and the shape parameter, *x*_b_, were treated as tuning parameters that were adjusted to understand how flow characteristics influence the chemical concentrations and the microbial populations present in venting fluids. While *Q′*_*vt*_ sets the timescale of fluid flow, the shape parameter, *x*_b_, determined the relative amount of time spent at various temperatures along the flow path. All model parameters and boundary conditions are provided as Supplementary Material.

The model state variables were concentration of CH_4_, [CH_4_] (μmol kg^−1^), concentration of H_2_, [H_2_] (μmol kg^−1^), concentration of a thermophilic methanogen with *M. thermolithotrophicus* growth kinetics, [*M*_*the*_] (cells L^−1^), concentration of a hyperthermophilic methanogen with *M. jannaschii* growth kinetics, [*M*_*jan*_] (cells L^−1^), concentration of Mg^2+^, [Mg^2+^] (mmol kg^−1^), and temperature, *T* (K). Temperature was calculated assuming pure mixing and was given by the following equation,

2 $$T = \frac{{f_{{\mathrm{ht}}}C_{{\mathrm{p,ht}}}T_{{\mathrm{ht}}} + \left( {1 - f_{{\mathrm{ht}}}} \right)C_{{\mathrm{p,sw}}}T_{{\mathrm{sw}}}}}{{f_{{\mathrm{ht}}}C_{{\mathrm{p,ht}}} + \left( {1 - f_{{\mathrm{ht}}}} \right)C_{{\mathrm{p,sw}}}}}$$

where *f*_ht_ is the fraction of high-temperature fluid in the box determine from the Mg^2+^ content of the venting fluid assuming 0 mmol Mg^2+^ kg^−1^ in the hydrothermal end-member, *T*_ht_ is the temperature of the hydrothermal end-member fluid, *T*_sw_ is the temperature of seawater, and *C*_p,ht_ and *C*_p,sw_ are the heat capacities of hydrothermal end-member fluid and seawater, respectively. During hydrothermal circulation, Mg^2+^ is removed from solution within hours at high temperatures and the Mg^2+^ content of diffuse fluids indicates how much hot, zero-Mg^2+^ end-member is in the fluid [29].

The rest of the variables were described by the following system of differential equations for each box *i*, which were solved using the method-of-lines. The model was implemented in the R software environment using the package ReacTran [30].

3 $$\frac{{d[{\mathrm{H}}_2]_i}}{{dt}} = - \frac{{\Delta _i\left( {Q \cdot \left[ {{\mathrm{H}}_2} \right]} \right)}}{{\Delta V_i}} + \frac{{Q_{sw} \cdot \left[ {{\mathrm{H}}_2} \right]^{sw}}}{{\Delta V_i}} - 4\mathop {\sum }\limits_{i = 1}^2 R_{{\mathrm{CH4}}_{i,j}}$$

4 $$\frac{{d[{\mathrm{CH}}_4]_i}}{{dt}} = - \frac{{\Delta _i(Q \cdot \left[ {{\mathrm{CH}}_4} \right])}}{{\Delta V_i}} + \frac{{Q_{sw} \cdot [{\mathrm{CH}}_4]^{sw}}}{{\Delta V_i}} + \mathop {\sum }\limits_{i = 1}^2 R_{{\mathrm{CH}}4_{i,j}}$$

5 $$\frac{{d[M_{{\mathrm{the}}}]_i}}{{dt}} = - \frac{{\Delta _i(Q \cdot \left[ {M_{{\mathrm{the}}}} \right])}}{{\Delta V_i}} + R_{M_{{\mathrm{the}}_i}}$$

6 $$\frac{{d[M_{{\mathrm{jan}}}]_i}}{{dt}} = - \frac{{\Delta _i(Q \cdot \left[ {M_{{\mathrm{jan}}}} \right])}}{{\Delta V_i}} + R_{M_{{\mathrm{jan}}_i}}$$

7 $$\frac{{d[{\mathrm{Mg}}]_i}}{{dt}} = - \frac{{\Delta _i(Q \cdot \left[ {{\mathrm{Mg}}} \right])}}{{\Delta V_i}} + \frac{{Q_{sw} \cdot [{\mathrm{Mg}}]^{sw}}}{{\Delta V_i}}$$

where *Q* is fluid flux. Methane production (*R*_CH4_) is calculated by:

8 $$R_{{\mathrm{CH}}4} = 10^{ - 9} \cdot v_{{\mathrm{max}}} \cdot \frac{{[{\mathrm{H}}_2]}}{{\left[ {{\mathrm{H}}_2} \right] + K_{{\mathrm{H}}_2}}} \cdot \frac{{e^{(T_{{\mathrm{max}}} - T)}}}{{1 + e^{(T_{{\mathrm{max}}} - T)}}}$$

where *v*_max_ is the maximum rate of cell-specific CH_4_ production, *K*_H2_ is the half-saturation constant for cell-specific CH_4_ production, and *T*_max_ is the optimum growth temperature of the methanogen. A conversion factor of 10^−9^ is used to convert *ν*_max_, which in Table S3 is expressed in terms of fmol cell^−1^ h^−1^ to μmol cell^−1^ h^−1^.

Growth rate for methanogens is given by,

9 $$R_{\mathrm{M}} = Ae^{ - \frac{{E_{\mathrm{a}}}}{{R_{\mathrm{g}}T}}} \cdot [{\mathrm{cells}}] \cdot \frac{{[{\mathrm{H}}_2]}}{{\left[ {{\mathrm{H}}_2} \right] + K_{{\mathrm{H}}_2}}} \cdot \frac{{e^{(T_{{\mathrm{max}}} - T)}}}{{1 + e^{(T_{{\mathrm{max}}} - T)}}}$$

where *A* is the Arrhenius constant and *E*_a_ is the activation energy.

### Field constraints on the reactive transport model

Field measurements of *Methanocaldococcus* species, *Methanothermococcus* species, CH_4_, and H_2_ concentrations in exiting fluids at individual diffuse vents were used to constrain the modeled subseafloor methanogen abundance, CH_4_ production, and the shape function of fluid mixing for each vent. Dissolved inorganic carbon (DIC) was also measured in the fluids to ensure that it was not growth limiting. Thirty-seven diffuse hydrothermal fluid samples were collected from Marker 113 and Marker 33 (Fig. 1) in 2013, 2014, and 2015 using the deep-sea remotely operated vehicles *Jason* II and *ROPOS* (Table S1) as previously described [8, 12]. The concentration of *Methanocaldococcus* and *Methanothermococcus* cells in the diffuse hydrothermal fluids was estimated from the product of the proportion of these organisms in annotated metagenomic sequence read counts [12] and the total cell counts [8] (Table S1).

There was no high-temperature hydrothermal venting within 0.5 km of Marker 113 or Marker 33 that could provide hydrothermal end-member gas concentrations. Therefore, high-temperature end-member H_2_ concentrations for these sites were estimated based on a trend of end-member H_2_ and end-member Cl^−^ for the closest high-temperature vents at Axial Seamount. The end-member Cl^−^ concentration of Marker 113 is ~100 mmol kg^−1^ and that of Marker 33 is ~ 400 mmol kg^−1^ (from extrapolation of diffuse fluid Cl^−^ to zero Mg^2+^). Temperature and chlorinity for the high-temperature end members were determined from the relationship of Mg^2+^ and temperature (or chlorinity) and extrapolated to zero Mg^2+^ concentration. The nearest high-temperature vents with similar Cl^−^ end-members are Diva vent (end-member Cl^−^ 200 mmol kg^−1^, end-member H_2_ 400–970 µmol kg^−1^) and El Guapo vent (end-member Cl^−^ 400 mmol kg^−1^ and end-member H_2_ 120–470 µmol kg^−1^). End-member H_2_ concentrations at Marker 113 and Marker 33 were assigned values of 950 and 300 μmol kg^−1^, respectively. These end-member H_2_ values for the diffuse vent sites reflect the fact that Marker 113 has a vapor-dominated source with higher gas content than the source for Marker 33.

## Results

### H_2_ Monod kinetics for methanogenesis

**Fig. 3:**
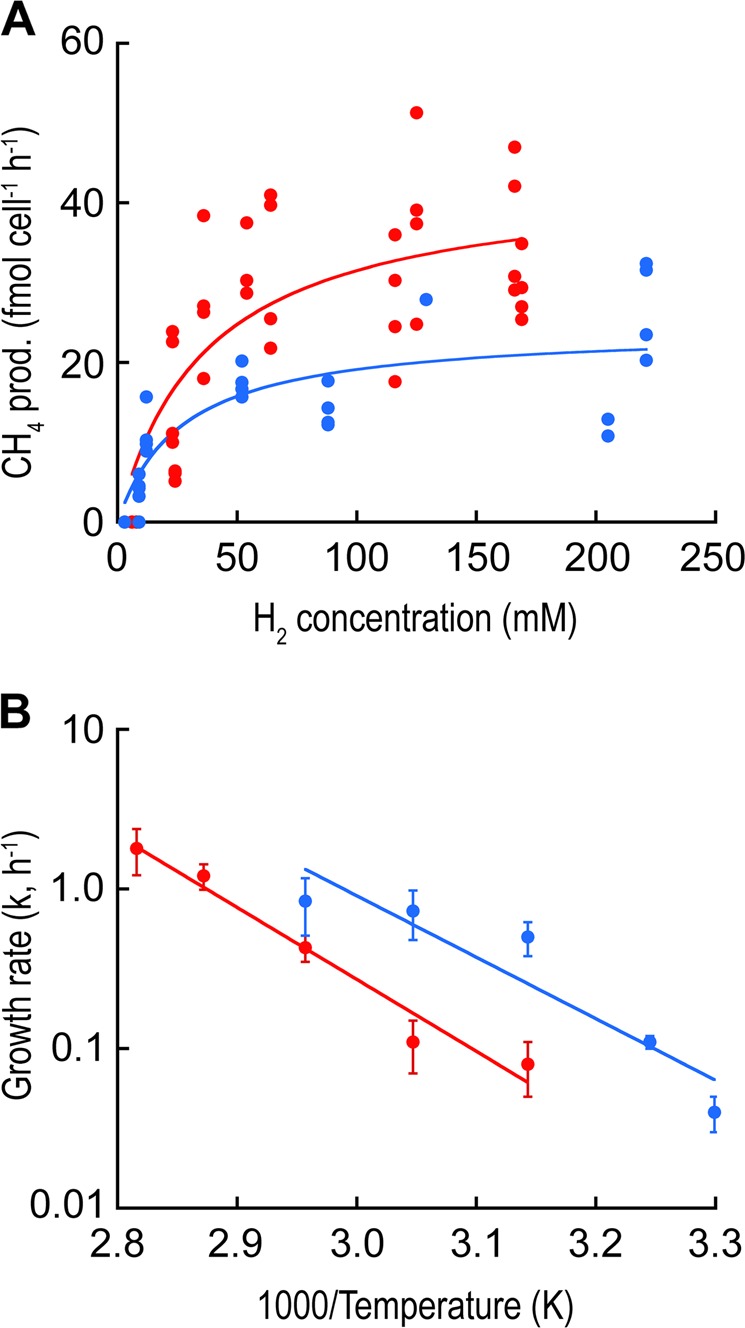
**a** Rates of methanogenesis as a function of H_2_ concentration and **b** growth rates as a function of 1/*T* for *M. jannaschii* (red solid circle) and *M. thermolithotrophicus* (blue solid circle). Monod kinetic parameters (*K*_H2_, *v*_max_) were determined from the data in plot (**a**) and the Arrhenius parameters (*A*, *E*_a_) were determined from the data in plot (**b**). The error bars in panel (**b**) represent 95% confidence intervals

*M. jannaschii* was grown in 11 separate chemostat runs and *M. thermolithotrophicus* in 9 separate chemostat runs at varying H_2_ concentrations, dilution rates, and temperatures (Table S2). The cell-specific CH_4_ production rates were not distinguishable based on dilution rate or growth temperature at each H_2_ concentration examined, so the Monod kinetics data were pooled for each organism (Fig. 3a). For *M. jannaschii*, the maximum CH_4_ production rate (*V*_max_) was 43 ± 5 fmol CH_4_ cell^−1^ h^−1^ (± standard error) and the *K*_S_ for H_2_ was 37 ± 15 µM. For *M. thermolithotrophicus*, the *V*_max_ was 24 ± 3 fmol CH_4_ cell^−1^ h^−1^ and the *K*_S_ for H_2_ was 27 ± 12 µM. The growth rates of both organisms in a closed system (Balch tubes) were measured over their growth temperature range to determine their Arrhenius constants (Table S3). For *M. jannaschii*, the activation energy (*E*_a_) was 86.3 kJ mol^−1^ and the pre-exponential factor (*A*) was 9.16 × 10^12^ h^−1^. For *M. thermolithotrophicus*, the *E*_a_ was 73.8 kJ mol^−1^ and *A* was 3.36 × 10^11^ h^−1^ (Fig. 3b).

### Reactive transport modeling

The parameter *Q′*_*vt*_ set the timescale or average residence time of fluid circulation through the hydrothermal system. If this parameter was large, then the residence time was short and there was not enough time spent at optimal growth conditions for a population to develop. Any microbes that might be present were simply washed out and H_2_ and CH_4_ followed conservative mixing between high-temperature source fluid and seawater. If *Q′*_*vt*_ was small, then the residence time was long and there was enough time for methanogen populations to establish in the fluid during its transport through the system. Any H_2_ present in the fluid was converted to CH_4_, producing significant H_2_ and CH_4_ anomalies from conservative mixing.

**Fig. 4:**
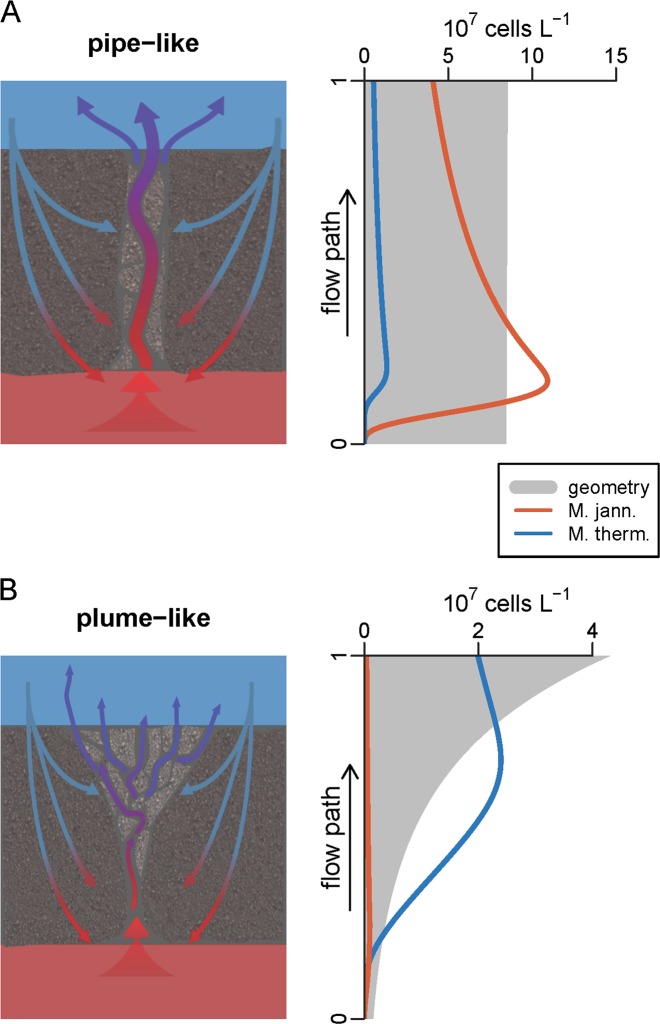
General reactive transport model results for straight-pipe (**a**) and expanding-plume (**b**) models. Lateral cross-sections depicting each model are shown on the left. The estimated concentration of *M. jannaschii* and *M. thermolithotrophicus* cells and the geometry of the flow path are shown on the right. The fluid temperatures at steps 0 and 1 are 84.6 and 26.7 °C, respectively

The pipe-like and expanding-plume models shown in Fig. 4 had identical final H_2_ and CH_4_ concentrations (Fig. S1), but the ratio of hyperthermophilic-to-thermophilic methanogens varied significantly. For fluid flow through a straight-pipe-like model, the hyperthermophile *Methanocaldococcus* dominated the system by consuming all the H_2_ prior to any significant growth of the thermophile *Methanothermococcus* (Fig. 4a). Fluid flow was constrained by the walls of the pipe, so as seawater was entrained into the pipe, mass conservation accelerated the flow, leaving the thermophiles more sensitive to washout than the hyperthermophiles. Alternatively, if the cross-sectional area increases along the vertical flow path, as in the expanding plume model, the thermophile *Methanothermococcus* dominated the system (Fig. 4b). The exact transition between the dominance of the two types of methanogens depended on the residence time, composition, and temperature of the end-member fluids.

The reactive transport model was applied to two sites of diffuse venting at Axial Seamount. The aqueous H_2_ and CH_4_ concentrations in exiting diffuse fluids were 0.1–2.6 μmol kg^−1^ of fluid and 13–40 μmol kg^−1^ of fluid at Marker 33 and Marker 113, respectively, indicating H_2_ consumption and CH_4_ production relative to conserved end-member mixing [12]. The DIC concentrations were 4.5–15.1 mmol kg^−1^ of fluid at these sites and were not considered limiting to methanogenesis. At Marker 113, 15–31% of annotated metagenomic sequences were assigned to known methanogenic genera, primarily thermophilic *Methanothermococcus* species, based on metagenomic and culture-dependent analyses (Table S1) [8, 12]. The estimated concentrations of *Methanothermococcus* and *Methanocaldococcus* cells in exiting vent fluids based on metagenomic analyses were 1.9 ± 0.7 × 10^8^ cells L^−1^ (± standard error) and 2.9 ± 0.9 × 10^7^ cells L^−1^, respectively. In contrast, at Marker 33, only 2–5% of annotated metagenomic sequences were assigned to known methanogenic genera, primarily hyperthermophilic *Methanocaldococcus* species (Table S1) [8, 12]. The estimated concentrations of *Methanothermococcus* and *Methanocaldococcus* cells in these exiting vent fluids, based metagenomic analyses, were 1.2 ± 0.3 × 10^6^ and 1.2 ± 0.3 × 10^7^ cells L^−1^, respectively.

**Fig. 5:**
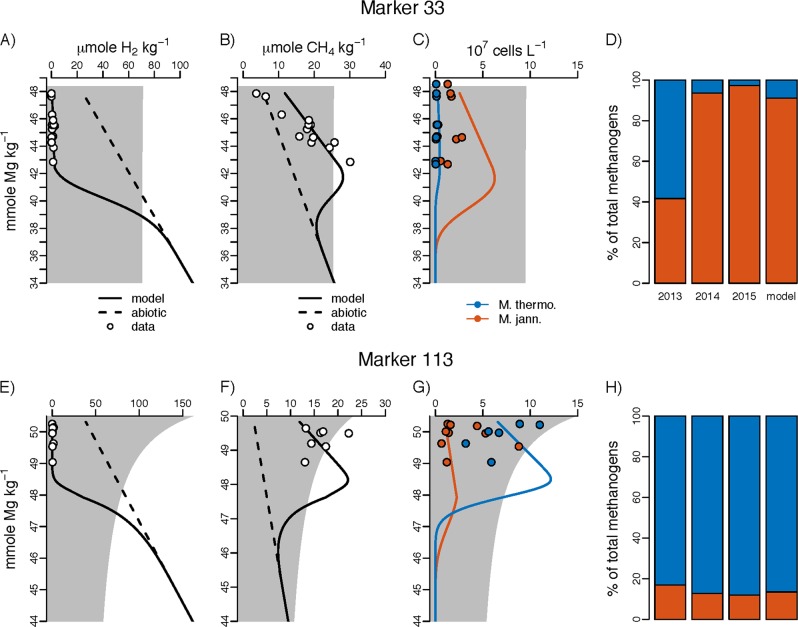
Field data and reactive transport model results for Marker 33 and Marker 113 at Axial Seamount. Model fits compared to field observations from 2013, 2014, and 2015 for Marker 33 (top row, **a**–**d**) and Marker 113 (bottom row, **e**–**h**). The first column (**a**, **e**) shows H_2_ concentrations vs. the conservative tracer Mg^2+^. The solid line shows the model fit and the dashed line the conservative abiotic mixing line. The shaded gray area shows the geometry of the mixing zone needed to produce the fit. The second column (**b**, **f**) shows CH_4_ concentrations vs. Mg^2+^. The third column (**c**, **g**) shows estimated methanogen cell concentrations from field measurements for the thermophilic (blue solid circle) and hyperthermophilic (red solid circle) methanogens and the modeled cell abundances. The fourth column (**d**, **h**) shows how the methanogen population is divided between thermophile and hyperthermophiles as estimated from the metagenomic data in Fortunato et al. [12] for each of the years compared to the model fit over all years

To fit the model, both the fluid flux (*Q′*_*vt*_) and shape parameter (*x*_b_) were adjusted until the H_2_ and CH_4_ concentrations and ratio of thermophiles to hyperthermophiles fit those observed in the vent fluid outflow (Fig. 5). The model closely reproduced the H_2_ and CH_4_ concentrations observed and methanogens estimated in the vent fluid outflow. For Marker 113 and Marker 33, the residence times of fluid below the surface were 33 and 29 h, respectively. Parameter values and boundary conditions used for the simulations are provided in Table S4.

## Discussion

*Methanocaldococcus* and *Methanothermococcus* species almost exclusively use H_2_ and CO_2_ as their carbon and energy sources. Some *Methanothermococcus* spp. can use formate in lieu of H_2_ and CO_2_ [23, 31], but microcosm enrichments using diffuse fluids from Axial Seamount that were spiked with formate or acetate instead of H_2_ did not result in CH_4_ production at 55 or 80 °C [8]. These attributes make these methanogens amenable to modeling based on H_2_ availability. Previous Monod kinetics determined for *Methanocaldococcus* spp. in a batch reactor generally predicted which hydrothermal vent fluid chemistries could support the reproduction of these methanogens [6]. However, growth and CH_4_ production in a chemostat at constant H_2_ flux rates were needed to estimate biomass production and the biogeochemical impact of high-temperature methanogens in the subseafloor. This study expanded the temperature range of these measurements by including a *Methanothermococcus* species. The *K*_S_ and minimum H_2_ threshold for CH_4_ production measured in this study in the chemostat for *M. jannaschii* and *M. thermolithotrophicus* were slightly lower than the growth kinetic values measured previously for three *Methanocaldococcus* species grown in a batch reactor [6].

Comparing the fitted model domains for Marker 113 and Marker 33, the shape function for Marker 113 was more expanding plume-like while that for Marker 33 was more pipe-like. Although the average residence time of hydrothermal circulation was similar for both vents, the local residence time in the optimal growth ranges for *Methanothermococcus* and *Methanocaldococcus* differed due to the hydrology of the flow paths. The plume-like hydrology of Marker 113 resulted in a shorter residence time for hotter fluid compared to cooler fluid, favoring *Methanothermoccus*, while the pipe-like geometry of Marker 33 had a longer residence time at the hotter temperatures favoring *Methanocaldococcus*. This trend is similar to that observed for these two vent sites using metagenomic analyses, where mesophilic *Epsilonbacteraeota* were predominant at Marker 113 while thermophilic *Epsilonbacteraeota* were predominant at Marker 33 [12].

The reactive transport model was non-dimensional, which permitted estimates of residence times but not the extent or volume of the subsurface biotope associated with each vent. If fluid flux estimates are known, then the model could be rescaled to estimate the subsurface volume and methanogen abundance (details of these calculations are described in the Supplementary Material). While diffuse fluid flux measurements at Marker 113 and Marker 33 are not available, Pruis and Johnson [32] used a hydrologically-sealed sampler to estimate the diffuse fluid flux in the ASHES vent field at Axial Seamount (Fig. 1), ~2 km from Marker 113 and Marker 33, where fluids emanate from a collection of similarly small (<5 cm width) fractures. The fluid flux estimate was 48 m^3^ m^−2^ y^−1^. Assuming this fluid flux rate for Marker 113 and Marker 33, our model predicted that each vent hosts 0.4–1.3 × 10^11^ methanogen cells m^−2^ of vent seafloor surface area (Table S5). Assuming an effective porosity of 10–30% [32], the methanogens would occupy only 1.8–18 m^3^ of ocean crust m^−2^ of vent seafloor surface area (Table S5). From this, the depth below the seafloor at which methanogen growth begins can be estimated (see Supplemental Materials). For Marker 33 where *x*_b_ = 100, methanogen growth begins 1.8–18 mbsf depending upon the porosity. For Marker 113 where *x*_b_ = 1, methanogen growth begins somewhere between 3 and 28 mbsf (Fig. S2). This result was consistent with previous field observations suggesting that the subsurface populations feeding these vents are predominately local features [12, 22, 33] drawing on nearby subsurface microbial populations, rather than large-scale subsurface hydrothermal fluid circulation patterns. The degree to which these local hot spots of microbial activity represent the overall subsurface biotope of Axial Seamount is unknown.

The 29–33 h subseafloor residence times of the diffuse fluids at Axial Seamount were comparable to the 17–41 h resident time estimate for microbes in a diffuse vent Crab Spa at 9°50ʹ East Pacific Rise hydrothermal vent site [3]. We similarly found that a relatively small but highly productive microbial standing stock in the subseafloor at vents likely accounts for the biogeochemical alteration and biomass output from subseafloor vent chemoautotrophs. Unlike Crab Spa where there was net CH_4_ consumption in the diffuse fluids [3], there was net biogenic CH_4_ production in our two diffuse vent fluids at Axial Seamount, demonstrating that in certain circumstances methanogens can represent a significant proportion of the total primary productivity.

One possible caveat for our model was that it only considered microbial growth in the fluid phase and not growth of microbes attached to surfaces. Hyperthermophiles, including *M. jannaschii*, produce biofilms and form attachments to hydrothermal minerals [34, 35]. The microbial mat and centimeter-scale flocculent material that was flushed from the seafloor immediately following a volcanic eruption at Axial and elsewhere [10, 36, 37] suggests that subsurface microbes attach to one another and to solid surfaces, most likely to prevent washout from the system. Incorporating biofilms into the model introduces additional parameters, such as the attachment strength and surface area available for colonization. For the Marker 113 model, the CH_4_ anomaly observed in the diffuse fluid matched the concentration of methanogens present in the vent outflow. This suggests that the methanogen population at this vent could exist in the fluid phase given the estimated residence times of the fluid at thermophilic growth temperatures. In contrast, the number of hyperthermophilic methanogens needed to produce the modeled CH_4_ anomaly at Marker 33 was much higher than the numbers observed in the diffuse fluid outflow (Fig. 4b). The ‘missing’ hyperthermophilic methanogens could be living in a subsurface biofilm.

Another possible caveat for our model is that it assumes a constant growth yield (i.e., amount of cell mass produced per mol of CH_4_ produced) across varying H_2_ concentrations and temperatures. In this study, *M. jannaschii* and *M. thermolithotrophicus* were grown in the chemostat at maximum cell concentrations such that all the available H_2_ in the reactor was consumed, which closely resembles the complete depletion of H_2_ in diffuse vent fluids exiting the seafloor. However, when *M. jannaschii* was grown in a chemostat at 80–83 μM H_2_ and 10-fold lower cell concentrations such that excess H_2_ was observed in the headspace, the growth yield (*Y*_p/CH4_) dropped ~10-fold and the cell-specific CH_4_ production rate increased to ~ 500 fmol CH_4_ cell^−1^ h^−1^ [38]. Growth yields also increased in laboratory studies in the hydrogenotrophic thermophile *Methanothermobacter thermoautotrophicus* and the mesophile *Methanococcus maripaludis* when H_2_ was limiting [39–41]. Therefore, the ‘missing’ hyperthermophilic methanogens at Marker 33 may also be due to excess H_2_ availability and low hyperthermophilic methanogen growth yields at hyperthermophilic temperatures that lead to much higher CH_4_ production rates per cell. In this study, the cell-specific CH_4_ production rates are conservative and the estimated number of total methanogens at each Axial vent in this study should be considered upper limits. This means that the population of methanogen cells necessary to support substantial methane anomalies could be even smaller than we have predicted.

## Conclusion

This study used two representative methanogens as tracers of subsurface fluid circulation and microbial production at two diffuse vents at Axial Seamount. By combining laboratory-derived methanogen growth kinetics with reactive transport modeling and in situ observations, the population size of methanogens, the volume of crust occupied by these organisms, the fluid residence time, and the nature of subsurface mixing were estimated. Results suggest that the methanogen population at each vent was relatively small and local, occupying as little as 2 m^3^ of subsurface crust and consisting of 0.4–1.3 × 10^11^ total methanogen cells m^−2^ of vent area. The model showed that the differences in the methanogen populations at Marker 113 and Marker 33 can be explained by differences in the geometry of the subsurface hydrology. Therefore, small-scale variation in the geologic fabric of the upper crust can create varied fluid flow paths fixed in space that harbor persistent and distinct microbial communities, whose metabolic activity produces microbial and chemical signatures at seafloor vents.

## Supplementary information

Supplemental Material
