# Supplementary material for: Fluid geochemistry, local hydrology, and metabolic activity define methanogen community size and composition in deep-sea hydrothermal vents
Source: ISME J. 2019 Mar 6;13(7):1711–21. doi: 10.1038/s41396-019-0382-3 (PMC6776001; doi:10.1038/s41396-019-0382-3)
Supplement: Supplementary file 1 — Supplemental Material [file 41396_2019_382_MOESM1_ESM.pdf]

Supplemental Material for

Fluid Chemistry, Local Hydrology, and Metabolic Activity Define Methanogen Community Size and Composition in Deep-Sea Hydrothermal Vents

Lucy C. Stewart[1]§, Christopher K. Algar[2], Caroline S. Fortunato[3], Benjamin I. Larson[4], Joseph J. Vallino[5], Julie A. Huber[f], David A. Butterfield[4], and James F. Holden[1]*

[1]*Department of Microbiology, University of Massachusetts, Amherst, MA, 01003, USA.*
[2]*Department of Oceanography, Dalhousie University, Halifax, B3H 4R2, Canada.*
[3]*Department of Biology, Wilkes University, Wilkes-Barre, PA 18766, USA.*
[4]*Joint Institute for the Study of Atmosphere and Ocean, University of Washington, Seattle, WA 98195, USA.*
[5]*Ecosystems Center, Marine Biological Laboratory, Woods Hole, MA, 02543, USA.*
[f]*Marine Chemistry and Geochemistry, Woods Hole Oceanographic Institution, Woods Hole, MA 02543, USA.*

§Current address: GNS Science, Wellington 5010, New Zealand.

*For correspondence. E-mail: jholden@microbio.umass.edu; Tel. (+1) 413 577 1742

This file includes:

- Methods for rescaling vent model results and estimating total cells in the system
- Fig. S1 and S2
- Tables S1 to S5
- References for SI reference citations

1.1 Re-scale vent model results

Pruis and Johnson [1] reported the yearly fluid flux from a 1 m^2 area of the ASHES vent field to be 43 m^3 m^{-2} y^{-1} and was equivalent to the Darcy flux (q_d). The Darcy flux is related to porewater velocity according to:

$$v = \frac{q_d}{\varphi} \tag{S1}$$

Therefore, flux of fluid through the pore spaces over a m^2 of seafloor was obtained by multiplying this flux by the porosity. For a porosity, φ, range 0.1-0.3, typical of young basalt crust, this equated to a flux of 0.018-0.054 m^3 m^{-2} h^{-1}. Assuming the flux is similar at Marker 113 and Marker 33, this was used to rescale the model results, according to:

$$Q'_{vt} = \frac{Q_{vt}}{V_{vt}} \tag{S2}$$

where Q'_{vt} is the timescale of hydrothermal circulation from the model fit, Q_{vt} is the observed fluid flux at the vent outflow, a V_{vt} is the actual subsurface volume of the hydrothermal mixing zone feeding the vent.

1.2 Calculating the total cells

The true volume of each grid cell was calculated by multiplying each grid cell by the true volume of the entire biosphere.

$$\Delta V_i = \Delta V'_i \cdot V_{tot} \cdot 1000 \tag{S3}$$

The factor of 1000 was used to convert m^3 to L since the model concentrations were in L. From this, the cell concentrations were integrated, according to:

$$\text{total cells} = \int [\text{cells}] dV \approx \sum_i^n [\text{cells}]_i \cdot \Delta V_i \tag{S4}$$

1.3 Maximum biosphere depth for Marker 113

Assume the shape function (Eq. 1) describes the cross-sectional area, and the *x* coordinate has been transformed so that χ=0 at the seafloor and χ=1 at the base of the model domain.

$$V = \int_0^{\frac{x_b}{\chi_b}} A_0 e^{-\frac{x'}{x_b}} dx'$$

$$V = -A_0 x_b e^{\frac{-x'}{x_b}} \Big]_0^{\frac{x_b}{\chi_b}}$$

$$V = -A_0 x_b e^{\frac{-1}{\chi_b}} + A_0 x_b$$
$$V = A_0 x_b \left(1 - e^{\frac{-1}{\chi_b}}\right)$$
$$x_b = \frac{V}{A_0\left(1 - e^{\frac{-1}{\chi_b}}\right)}$$

Marker 113: χ_b = 1, Vol = 18 m^2, Area = 1 m^2

$$x_b = \frac{18}{1 \cdot \left(1 - \frac{1}{e}\right)}$$

$$x_b = 28.4 \text{ m}$$

And the onset of methanogenesis occurs at a depth of $\frac{1}{\chi_b} x_b = \left(\frac{1}{1}\right) \cdot 28.4 = 28.4\ m$

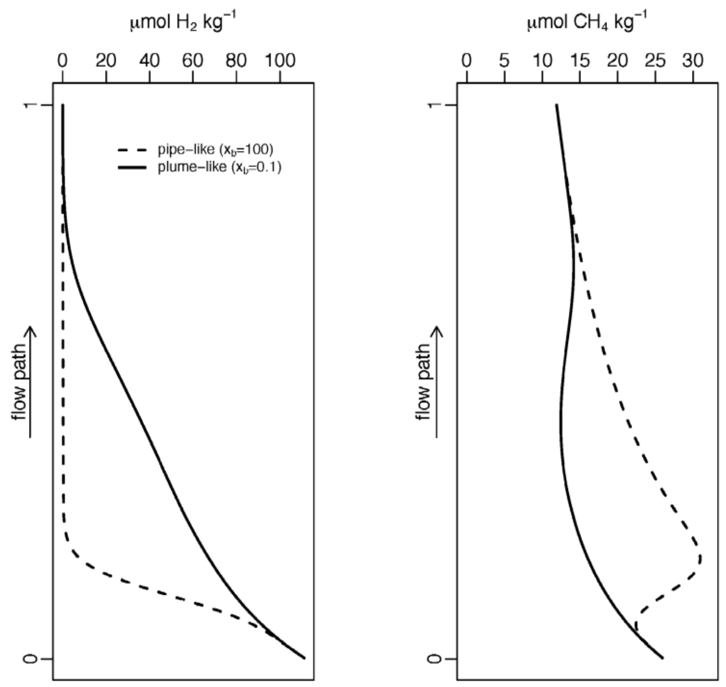

Fig. S1. General reactive transport model results H_2 (left) and CH_4 (right) for straight-pipe and expanding-plume models. The fluid temperatures at steps 0 and 1 are 84.6°C and 26.7°C, respectively.

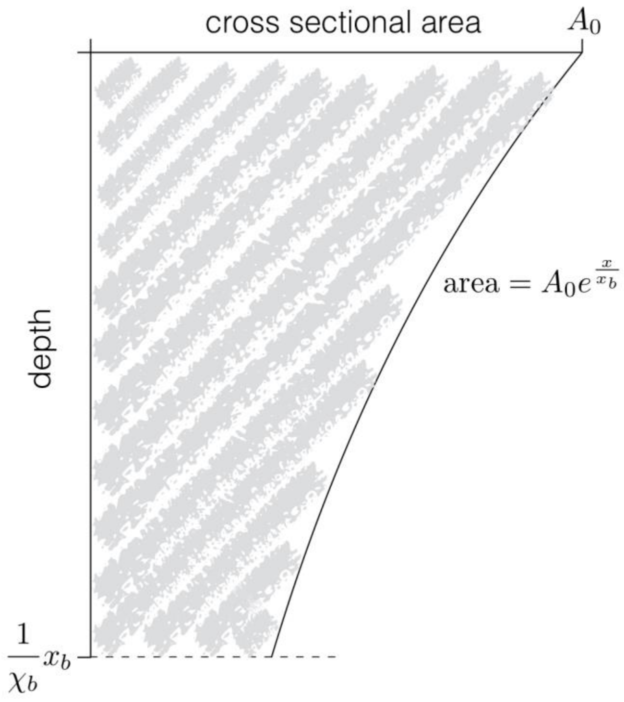

Fig S2. Cross sectional area for the true model domain vs depth. A_0 is the true area of venting at the seafloor. x_b is the non-dimensional shape parameter from Eq. 1. x_b is the true shape parameter with units of length. The quantity $\frac{1}{\chi_b} x_b$ is the depth below the seafloor where methanogen growth starts.

Table S1. Fluid chemistry, total cell concentrations, and cell concentration estimates for hyperthermophilic (H) and thermophilic (T) methanogens based on their proportions of total cells for Marker 113 and Marker 33 vents (from 2, 3)

Sample ID	Date	Temp. (°C)	$[Mg^{2+}]$ (mmol kg^{-1})	[total cell] (ml^{-1})	$[\text{methanogen}]_H$ (ml^{-1})	$[\text{methanogen}]_T$ (ml^{-1})	$[H_2]$ (μmol kg^{-1})	$[CH_4]$ (μmol kg^{-1})
**Marker 113:**								
Metagenome	9/27/2013				2.5%	12.3%		
R1663-HFS-01	9/27/2013	24.1	50.34	-			< 1	13.240
R1663-HFS-02	9/27/2013	24.0	49.82	2.63×10^5	6.5×10^3	3.2×10^4	1.350	14.400
R1663-HFS-04	9/27/2013	23.5	49.18	4.76×10^5	1.2×10^4	5.9×10^4	< 1	13.050
R1663-HFS-07	9/27/2013	24.4	50.19	5.45×10^5	1.4×10^4	6.7×10^4	< 1	16.333
R1663-HFS-08	9/27/2013	24.5	50.23	4.56×10^5	1.1×10^4	5.6×10^4	< 1	16.867
Metagenome	8/12/2014				1.9%	13.1%		
J786-HFS-01	8/12/2014	24.3	50.49	6.80×10^5	1.3×10^4	8.9×10^4	0.004	34.836
J786-HFS-03	8/12/2014	25.1	50.46	8.50×10^5	1.6×10^4	1.1×10^5	-	-
J786-HFS-08	8/12/2014	24.9	50.42	2.30×10^6	4.4×10^4	3.0×10^5	-	-
J786-IGT-11	8/12/2014	26.4	50.37	-	-	-	1.700	36.700
J786-IGT-12	8/12/2014	27.4	50.35	-	-	-	0.600	40.300
Metagenome	8/25/2015				3.5%	25.9%		
J824-HFS-13	8/25/2015	25.4	49.72	2.50×10^6	8.8×10^4	6.5×10^5	-	-
J824-HFS-14	8/25/2015	25.2	49.72	-	-	-	0.390	17.443
J824-HFS-18	8/25/2015	25.4	50.17	1.50×10^6	5.3×10^4	3.9×10^5	0.240	22.270
**Marker 33:**								
Metagenome	9/28/2013				0.5%	0.7%		
R1665-HFS-01	9/28/2013	27.6	45.80	-	-	-	2.600	53.530
R1665-HFS-02	9/28/2013	28.3	45.55	-	-	-	< 1	17.996
R1665-HFS-03	9/28/2013	27.8	44.97	2.95×10^5	1.5×10^3	2.1×10^3	1.231	15.824
R1665-HFS-05	9/28/2013	27.3	45.89	4.17×10^5	2.1×10^3	2.9×10^3	< 1	18.987
R1665-HFS-06	9/28/2013	26.7	45.89	2.79×10^5	1.4×10^3	2.0×10^3	1.850	18.400
R1665-IGT-08	9/28/2013	39.4	42.96	-	-	-	1.310	30.122
R1665-HFS-09	9/28/2013	27.0	46.69	-	-	-	< 1	10.850

Sample ID	Date	Temp. (°C)	$[Mg^{2+}]$ (mmol kg^{-1})	[cell] (ml^{-1})	$[methanogen]_H$ (ml^{-1})	$[methanogen]_T$ (ml^{-1})	$[H_2]$ (μmol kg^{-1})	$[CH_4]$ (μmol kg^{-1})
**Marker 33 (cont.):**								
Metagenome	8/16/2014				4.5%	0.3%		
J790-HFS-05	8/16/2014	19.0	49.10	2.95×10^5	1.3×10^4	885	-	-
J790-HFS-06	8/16/2014	18.5	48.12	3.85×10^5	1.7×10^4	1.2×10^3	0.033	6.357
J790-HFS-07	8/16/2014	18.7	48.36	3.50×10^5	1.6×10^4	1.1×10^3	0	3.697
J790-IGT-11	8/16/2014	21.3	46.25	-	-	-	1.300	18.500
J790-IGT-12	8/16/2014	31.8	44.09	-	-	-	1.600	24.300
Metagenome	8/21/2015				3.5%	0.1%		
J822-HFS-05	8/21/2015	33.1	44.75	6.30×10^5	2.2×10^4	630	-	-
J822-HFS-06	8/21/2015	33.0	44.49	-	-	-	0.092	19.215
J822-HFS-07	8/21/2015	34.0	44.90	8.10×10^5	2.8×10^4	810	0.136	19.721
J825-HFS-05	8/26/2015	40.6	44.50	-	-	-	0.132	25.700
J825-HFS-06	8/26/2015	40.5	43.03	1.57×10^5	5.5×10^3	157	-	-
J825-HFS-07	8/26/2015	40.3	42.78	3.60×10^5	1.3×10^4	360	-	-

Table S2. Chemostat data for *Methanocaldococcus jannaschii* and *Methanothermococcus thermolithotrophicus*

$[H_2]$ (μM)	D (h^{-1})	Dilution rate (ml min^{-1})	[Cell] (ml^{-1})	Cell prod. rate (cells h^{-1})	Liquid $[CH_4]$ (nmol ml^{-1})	Headspace $[CH_4]$ (μmol ml^{-1})	CH_4 prod. rate (mmol h^{-1})	Specific CH_4 prod. rate (fmol $cell^{-1}$ h^{-1})	Cell yield (cells × 10^{12} mol CH_4^{-1})
***M. jannaschii* (82°C):**									
166	0.576	14.4	1.30×10^{8}	1.12×10^{11}	50.2	0.939	5.67	29.1	19.8
166	0.576	14.4	1.22×10^{8}	1.05×10^{11}	62.0	0.930	5.63	30.8	18.7
166	0.508	12.7	9.80×10^{7}	7.47×10^{10}	46.4	1.146	6.91	47.0	10.8
166	0.508	12.7	1.10×10^{8}	8.38×10^{10}	46.8	1.151	6.94	42.1	12.1
116	0.519	13.0	7.80×10^{7}	6.08×10^{10}	38.7	0.836	3.54	30.3	17.2
116	0.519	13.0	7.20×10^{7}	5.61×10^{10}	36.8	0.918	3.89	36.0	14.4
116	0.454	11.3	5.00×10^{7}	3.40×10^{10}	35.5	0.432	1.84	24.5	18.5
116	0.454	11.3	6.60×10^{7}	4.49×10^{10}	40.7	0.408	1.74	17.6	25.8
64	0.374	9.4	2.60×10^{7}	1.46×10^{10}	17.8	0.379	1.60	41.0	9.1
64	0.374	9.4	2.60×10^{7}	1.46×10^{10}	17.6	0.366	1.55	39.7	9.4
64	0.295	7.4	3.40×10^{7}	1.50×10^{10}	19.6	0.307	1.30	25.5	11.5
64	0.295	7.4	3.80×10^{7}	1.68×10^{10}	19.5	0.294	1.24	21.8	13.5
23	0.398	10.0	3.00×10^{7}	1.79×10^{10}	7.3	0.242	1.02	22.6	17.5
23	0.398	10.0	2.80×10^{7}	1.67×10^{10}	8.9	0.238	1.01	23.9	16.5
23	0.356	8.9	3.00×10^{7}	1.60×10^{10}	10.3	0.106	0.45	10.0	35.6
23	0.356	8.9	2.90×10^{7}	1.55×10^{10}	7.8	0.114	0.48	11.1	32.3
6	0	0	NG	NG	ND	ND	0	0	0
6	0	0	NG	NG	ND	ND	0	0	0
***M. jannaschii* (65°C):**									
169	0.553	13.8	4.20×10^{7}	3.48×10^{10}	54.3	0.359	2.17	34.9	16.0
169	0.553	13.8	5.20×10^{7}	4.31×10^{10}	53.2	0.344	2.11	27.0	20.4
169	0.438	11.0	5.40×10^{7}	3.55×10^{10}	84.0	0.387	2.38	29.4	14.9
169	0.438	11.0	6.00×10^{7}	3.95×10^{10}	85.8	0.371	2.28	25.4	17.3
125	0.504	12.6	2.60×10^{7}	1.97×10^{10}	44.9	0.355	1.53	39.1	12.9
125	0.504	12.6	2.00×10^{7}	1.51×10^{10}	44.7	0.358	1.54	51.3	9.8

$[H_2]$ (µM)	D (h^{-1})	Dilution rate (ml min^{-1})	[Cell] (ml^{-1})	Cell prod. rate (cells h^{-1})	Liquid $[CH_4]$ (nmol ml^{-1})	Headspace $[CH_4]$ (µmol ml^{-1})	CH_4 prod. rate (mmol h^{-1})	Specific CH_4 prod. rate (fmol $cell^{-1}$ h^{-1})	Cell yield (cells × 10^{12} mol CH_4^{-1})
***M. jannaschii* (65°C, cont.):**									
125	0.396	9.9	3.00×10^7	1.78×10^{10}	42.4	0.395	1.69	37.4	10.5
125	0.396	9.9	4.80×10^7	2.85×10^{10}	45.5	0.419	1.79	24.8	15.9
54	0.384	9.6	6.75×10^6	3.88×10^9	27.1	0.081	0.31	30.3	12.5
54	0.314	7.9	6.63×10^6	3.12×10^9	23.0	0.100	0.37	37.5	8.4
54	0.314	7.9	9.38×10^6	4.42×10^9	44.4	0.106	0.40	28.7	11.1
36	0.380	9.5	8.13×10^6	4.63×10^9	23.7	0.049	0.22	18.0	21.0
36	0.380	9.5	9.75×10^6	5.55×10^9	27.5	0.091	0.40	27.1	13.9
36	0.330	8.3	7.00×10^6	3.47×10^9	34.4	0.092	0.40	38.4	8.7
36	0.330	8.3	1.11×10^7	5.51×10^9	32.6	0.100	0.44	26.3	12.5
24	0.421	10.5	1.83×10^7	1.15×10^{10}	4.2	0.040	0.17	6.2	67.6
24	0.421	10.5	2.10×10^7	1.33×10^{10}	6.8	0.038	0.16	5.1	83.1
24	0.246	6.1	1.54×10^7	5.66×10^9	ND	0.034	0.14	6.1	40.4
24	0.246	6.1	1.86×10^7	6.86×10^9	ND	0.043	0.18	6.4	38.1
8	0	0	NG	NG	ND	ND	0	0	0
8	0	0	NG	NG	ND	ND	0	0	0
***M. thermolithotrophicus* (65°C):**									
221	0.495	12.4	4.40×10^7	3.27×10^{10}	28.9	0.491	2.08	31.6	15.7
221	0.495	12.4	6.20×10^7	4.60×10^{10}	25.1	0.444	1.88	20.3	24.5
221	0.431	10.8	5.60×10^7	3.62×10^{10}	26.3	0.645	2.72	32.4	13.3
221	0.431	10.8	7.40×10^7	4.78×10^{10}	24.6	0.618	2.61	23.5	18.3
129	0.576	14.4	1.10×10^8	9.50×10^{10}	9.7	1.092	4.60	27.9	20.7
88	0.670	16.8	6.40×10^7	6.44×10^{10}	17.7	0.281	1.20	12.5	53.7
88	0.670	16.8	6.20×10^7	6.23×10^{10}	17.1	0.267	1.14	12.2	54.6
88	0.403	10.1	5.40×10^7	3.26×10^{10}	15.7	0.339	1.43	17.7	22.8
88	0.403	10.1	6.60×10^7	3.99×10^{10}	13.7	0.334	1.41	14.3	28.3
12	0.447	11.2	3.60×10^7	2.41×10^{10}	7.1	0.114	0.48	8.9	50.2
12	0.447	11.2	2.40×10^7	1.61×10^{10}	4.6	0.134	0.56	15.7	28.6
12	0.275	6.9	3.80×10^7	1.57×10^{10}	5.8	0.133	0.56	9.8	28.0

$[H_2]$ (μM)	D (h^{-1})	Dilution rate (ml min^{-1})	[Cell] (ml^{-1})	Cell prod. rate (cells h^{-1})	Liquid $[CH_4]$ (nmol ml^{-1})	Headspace $[CH_4]$ (μmol ml^{-1})	CH_4 prod. rate (mmol h^{-1})	Specific CH_4 prod. rate (fmol $cell^{-1}$ h^{-1})	Cell yield (cells × 10^{12} mol CH_4^{-1})
***M. thermolithotrophicus* (65°C, cont.):**									
12	0.275	6.9	3.60×10^{7}	1.48×10^{10}	6.0	0.131	0.55	10.3	26.9
9	0	0	NG	NG	ND	ND	0	0	0
***M. thermolithotrophicus* (55°C):**									
205	0.720	18.0	8.20×10^{7}	8.85×10^{10}	122.4	0.345	1.59	12.9	55.7
205	0.720	18.0	8.60×10^{7}	9.28×10^{10}	138.2	0.280	1.40	10.8	66.3
205	0.461	11.5	1.44×10^{8}	9.96×10^{10}	110.3	0.230	1.00	4.6	99.6
205	0.461	11.5	1.24×10^{8}	8.57×10^{10}	191.7	0.255	1.11	6.0	77.2
52	0.674	16.9	3.00×10^{7}	3.03×10^{10}	28.5	0.175	0.79	17.5	38.4
52	0.674	16.9	3.00×10^{7}	3.03×10^{10}	30.7	0.167	0.75	16.7	40.4
52	0.315	10.5	3.80×10^{7}	2.39×10^{10}	42.5	0.269	1.15	20.2	20.8
52	0.315	10.5	4.80×10^{7}	3.02×10^{10}	38.8	0.265	1.13	15.7	26.7
9	0.428	10.7	2.20×10^{7}	1.41×10^{10}	5.7	0.018	0.11	3.2	128.2
9	0.428	10.7	2.00×10^{7}	1.28×10^{10}	3.2	0.021	0.13	4.3	98.5
9	0.282	7.1	2.13×10^{6}	9.00×10^{8}	0.4	0.003	0.02	6.0	45.0
9	0.282	7.1	2.88×10^{6}	1.22×10^{9}	1.3	0.003	0.02	4.5	61.0
3	0	0	NG	NG	ND	ND	0	0	0

NG, no growth; ND, not detected.
D = (Dilution rate × 60) ÷ 1,500 ml
Cell production rate = Dilution rate × [Cell] × 60
CH_4 production rate = ((Liquid $[CH_4]$ × Dilution rate) + (Headspace $[CH_4]$ × Gas flow rate)) × 60
Cell-specific CH_4 production rate (q) = CH_4 production rate ÷ ([Cell] × 1,500 ml)
Cell yield (Y_{CH4}) = Cell production rate ÷ CH_4 production rate

Table S3. Arrhenius growth rate data for *Methanocaldococcus jannaschii* and *Methanothermococcus thermolithotrophicus*

Growth condition	Growth rate (μ, h^{-1})
M. jannaschii:	
45°C	0.08 ± 0.03
55°C	0.11 ± 0.04
65°C	0.43 ± 0.08
75°C	1.21 ± 0.22
82°C	1.80 ± 0.58
84°C	1.10 ± 0.23
M. thermolithotrophicus:	
30°C	0.04 ± 0.01
35°C	0.11 ± 0.01
45°C	0.50 ± 0.12
55°C	0.73 ± 0.25
65°C	0.84 ± 0.33
68°C	0.82 ± 0.16

Table S4. Parameters, values, and boundary conditions used for simulations

Parameter	Mkr 113	Mkr 33	Units	Source
Transport parameters				
Vent fluid flux, Q_{vt}	0.030	0.035	h^{-1}	model fit
Shape parameter, x_b	1	100	-	model fit
Heat capacity of seawater, $C_{p,sw}$	4.143	4.143	kJ kg^{-1}°C^{-1}	calculated*
Heat capacity of HT end member, $C_{p,ht}$,	6.70	4.47	kJ kg^{-1}	calculated*
Growth kinetics for M. jannaschii				
Arrhenius constant, A	$9.12x10^{-12}$	$9.12x10^{-12}$	h^{-1}	chemostat
Activation energy, E_a	86.3	86.3	kJ	chemostat
H_2 half saturation, k_{H2}	37	37	µmol L^{-1}	chemostat
Maximum growth temperature, T_{max}	85	85	°C	chemostat
Cell specific CH_4 production, ν_{max}	43	43	fmol $cell^{-1}$ h^{-1}	chemostat
Growth kinetics for M. thermolithotrophicum				
Arrhenius constant, A	$3.36x10^{-11}$	$3.36x10^{-11}$	h^{-1}	chemostat
Activation energy, E_a	73.8	73.8	kJ	chemostat
H_2 half saturation, k_{H2}	27	27	µmol L^{-1}	chemostat
Maximum growth temperature, T_{max}	70	70	°C	chemostat
Cell specific CH_4 production, ν_{max}	24	24	fmol $cell^{-1}$ h^{-1}	chemostat
Boundary conditions				
H_2 in HT end member, $H_{2,ht}$	950	300	µmol kg^{-1}	estimate
H_2 in seawater, $H_{2,sw}$	0	0	µmol kg^{-1}	field data
CH_4 in HT end member	56	70	µmol kg^{-1}	estimate
CH_4 in seawater	0	0	µmol kg^{-1}	field data
Temperature of HT end member, T_{ht}	330	214	°C	estimate
Mg in HT end member, Mg_{ht}	0	0	mmol kg^{-1}	field data
Mg in seawater, Mg_{sw}	52.7	52.7	mmol kg^{-1}	field data
M. jannaschii in seawater	0	0	cells L^{-1}	
M. jannaschii in HT end member	1	1	cells L^{-1}	
M. thermolithotrophicum in seawater	0	0	cells L^{-1}	
M. thermolithotrophicum in HT end member	1	1	cells L^{-1}	

*Heat capacities are calculated using supcrt92 [4] implemented in the R package CHNOSZ [5].

Table S5. Reactive transport model results for methanogenesis at Marker 113 and Marker 33 vents at Axial Seamount

	Marker 113	Marker 33
Total biosphere volume[1,2]	2.0-18 m^3	1.8-16 m^3
Average fluid residence time	33 h	29 h
Total *M. thermolithotrophicum*	3.6×10^{10}-1.1×10^{11} cells	2.0×10^{9}-6.1×10^{9} cells
Total *M. jannaschii*	8.5×10^{9}-2.6×10^{10} cells	4.2×10^{10}-1.3×10^{11} cells
Total methanogens	4.5×10^{10}-1.3×10^{11} cells	4.4×10^{10}-1.4×10^{11} cells

[1]Estimated upward fluid velocities of 48 m^3 m^{-2} y^{-1} [1]

[2]Total biosphere volumes are estimated using a porosity range of 0.1-0.3

References

1. Pruis MJ, Johnson HP (2004) Tapping into the sub-seafloor: examining diffuse flow and temperature from an active seamount on the Juan de Fuca Ridge. *Earth Planet Sci Lett* 217:379-388.
2. Topçuoğlu BD, et al. (2016) Hydrogen limitation and syntrophic growth among natural assemblages of thermophilic methanogens at deep-sea hydrothermal vents. *Front Microbiol* 7:1240.
3. Fortunato CS, Larson B, Butterfield DA, Huber JA (2018) Spatially distinct, temporally stable microbial populations mediate biogeochemical cycling at and below the seafloor in hydrothermal vent fluids. *Environ Microbiol* 20:769-784.
4. Johnson JW, Oelkers EH, Helgeson HC (1992) SUPCRT92: a software package for calculating the standard molal thermodynamic properties of minerals, gases, aqueous species, and reactions from 1 to 5000 bar and 0 to 1000°C. *Comput. Geosci.* 18:899-947.
5. Dick JM (2008) Calculation of the relative metastabilities of proteins using the CHNOSZ software package. *Geochem. Trans.* 9:10.
